# Positive Selection and Functional Divergence at Meiosis Genes That Mediate Crossing Over Across the *Drosophila* Phylogeny

**DOI:** 10.1534/g3.119.400280

**Published:** 2019-07-30

**Authors:** Cara L. Brand, Lori Wright, Daven C. Presgraves

**Affiliations:** Department of Biology, University of Rochester, Rochester, New York, 14627

**Keywords:** recombination, crossing over, evolution, positive selection, *Drosophila*

## Abstract

Meiotic crossing over ensures proper segregation of homologous chromosomes and generates genotypic diversity. Despite these functions, little is known about the genetic factors and population genetic forces involved in the evolution of recombination rate differences among species. The dicistronic meiosis gene, *mei-217/mei-218*, mediates most of the species differences in crossover rate and patterning during female meiosis between the closely related fruitfly species, *Drosophila melanogaster* and *D. mauritiana*. The MEI-218 protein is one of several meiosis-specific mini-chromosome maintenance (mei-MCM) proteins that form a multi-protein complex essential to crossover formation, whereas the BLM helicase acts as an anti-crossover protein. Here we study the molecular evolution of five genes— *mei-218*, the other three known members of the mei-MCM complex, and *Blm*— over the phylogenies of three *Drosophila* species groups— *melanogaster*, *obscura*, and *virilis*. We then use transgenic assays in *D. melanogaster* to test if molecular evolution at *mei-218* has functional consequences for crossing over using alleles from the distantly related species *D. pseudoobscura* and *D. virilis*. Our molecular evolutionary analyses reveal recurrent positive selection at two mei-MCM genes. Our transgenic assays show that sequence divergence among *mei-218* alleles from *D. melanogaster*, *D. pseudoobscura*, and *D. virilis* has functional consequences for crossing over. In a *D. melanogaster* genetic background, the *D. pseudoobscura mei-218* allele nearly rescues wildtype crossover rates but alters crossover patterning, whereas the *D. virilis mei-218* allele conversely rescues wildtype crossover patterning but not crossover rates. These experiments demonstrate functional divergence at *mei-218* and suggest that crossover rate and patterning are separable functions.

During the early stages of meiosis, recombination occurs between homologous chromosomes, serving two functions. First, recombination repairs programmed DNA double-strand breaks (DSBs) and ensures proper Mendelian segregation of homologous chromosomes (Baker and Hall 1976; Lindsley and Sandler 1977). Second, recombination increases the efficacy of natural selection by reducing genetic linkage and creating novel genotypes (Fisher 1930; Felsenstein 1974; Crow 1992; Barton and Charlesworth 1998). Despite these benefits, recombination has risks. Dispersed selfish repetitive DNA sequences— *e.g.*, transposons— introduce the risk of non-homologous ectopic exchange that generates chromosomal duplications and deletions (Goldberg *et al.* 1983; Charlesworth *et al.* 1994). The rate and distribution of crossing over may therefore evolve to balance the benefits of recombination and the costs of ectopic exchange (Montgomery *et al.* 1987; Charlesworth and Barton 1996; Kent *et al.* 2017; Brand *et al.* 2018).

Recombination landscapes vary within and among taxa, but the genes, mechanisms, and evolutionary causes involved are still largely unknown (Ritz *et al.* 2017; Stapley *et al.* 2017). In mammals, four loci are associated with intraspecific variation in recombination rates: RNF212, CPLX1, REC8, and PRDM9 (Baudat *et al.* 2010; Sandor *et al.* 2012; Kong *et al.* 2014). Best studied is the gene, *Prdm9*, which encodes a *trans*-acting major determinant of recombination distribution in most mammals (Baudat *et al.* 2010; Myers *et al.* 2010; Parvanov *et al.* 2010). The PRDM9 protein binds specific DNA sequence motifs and modifies local histones, initiating the formation of DSBs nearby (Baudat *et al.* 2010; Myers *et al.* 2010). These DSBs are concentrated in the immediate vicinity of the motif, creating recombination “hotspots” once repaired. In rodents and primates, *Prdm9* shows signals of recurrent positive selection, particularly at sites encoding a zinc finger array that mediates DNA motif binding specificity (Oliver *et al.* 2009). The recurrent evolution at *Prdm9* alters the genomic distribution of recombination hotspots between closely related species and can, incidentally, cause sterility in species hybrids (Ptak *et al.* 2005; Davies *et al.* 2016; Smagulova *et al.* 2016).

In *Drosophila*, variation in the rate of recombination during female meiosis (males are achiasmate) exists along the lengths of chromosomes, among individuals, and between closely related species. Along chromosomes, rates of crossing over tend to be highest in medial euchromatic regions, lowest in centromere- and telomere-proximal regions (Dobzhansky 1930; Beadle 1932; Baker and Carpenter 1972; Lindsley and Sandler 1977), and absent in heterochromatic regions where repetitive DNA sequences are abundant (Baker 1958). Among individuals, natural genetic variation in crossover rates exists and responds to artificial selection (Kidwell 1972; Charlesworth and Charlesworth 1985; Brooks and Marks 1986; Brooks 1988; Comeron *et al.* 2012; Hunter *et al.* 2016). Between species, mean crossover frequencies vary more than two-fold (Ortiz-Barrientos *et al.* 2006; Comeron *et al.* 2012; L. Hemmer *et al*. unpublished). Despite these observations, few genetic loci are known that contribute to the observed variation in recombination rates (Hunter *et al.* 2016).

The genetic basis for recombination differs between flies and mammals. For one, many genes identified in mammals, including *Prdm9*, appear to be absent from *Drosophila* (Oliver *et al.* 2009; Heil and Noor 2012). Consistent with this, *Drosophila* lack comparably strong recombination hotspots (Comeron *et al.* 2012; Hunter *et al.* 2016). *Drosophila* also lack *Msh4* and *Msh5*, the canonical proteins that promote crossover formation in most eukaryotes. Instead, flies have co-opted a meiosis-specific mini-chromosome maintenance (mei-MCM) complex encoded by the genes *mei-217*, *mei-218*, *rec*, and (presumably) *Mcm5* to promote the formation of class I crossovers (Zalevsky *et al.* 1999; Kohl *et al.* 2012; Kohl and Sekelsky 2013). Class I crossovers are derived from heteroduplex DNA molecules (crossover intermediates) which are stabilized by the mei-MCM complex and ultimately resolved as crossover events (Figure 1; Kohl *et al.* 2012; Kohl and Sekelsky 2013; Hatkevich *et al.* 2017; Hatkevich and Sekelsky 2017). Class I crossovers are patterned by interference mechanisms that reduce the probability of a second crossover establishing nearby (Muller 1916) and by crossover suppression mechanisms that discourage crossover formation in telomere- and centromere-proximal regions (Dobzhansky 1930; Beadle 1932). Mutations in mei-MCM genes result in a >90% reduction in crossover frequency and a uniform chromosomal distribution of residual crossovers (Baker and Carpenter 1972; Carpenter and Sandler 1974; Grell 1984; Lake *et al.* 2007). These residual crossovers, termed class II crossovers, are uniformly distributed with chromosome length and thus lack the spatial patterning that results from crossover interference or telomere- and centromere-proximal crossover suppression (Figure 1; reviewed in Kohl and Sekelsky 2013). While the mei-MCMs promote class I crossover formation, the BLM helicase antagonizes crossover formation by dissolving heteroduplex DNA at multiple stages (Hatkevich and Sekelsky 2017). BLM unwinds the D-loops formed by strand invasion, leading to synthesis-dependent strand annealing (SDSA), the cause of most non-crossover gene conversion events (Figure 1; Allers and Lichten 2001). BLM also dissolves crossover intermediates in the class II pathway, as the mei-MCMs do not act to stabilize these (Figure 1; Kohl *et al.* 2012; Hatkevich *et al.* 2017; Hatkevich and Sekelsky 2017). Thus, BLM has anticrossover function, whereas the mei-MCMs are said to have “anti-anticrossover” function (Kohl and Sekelsky 2013). Given the antagonistic activities of the mei-MCM proteins and the BLM helicase, it seems plausible that molecular evolution at any of these proteins could contribute to phenotypic evolution of crossover rate and distribution.

**Figure 1:**
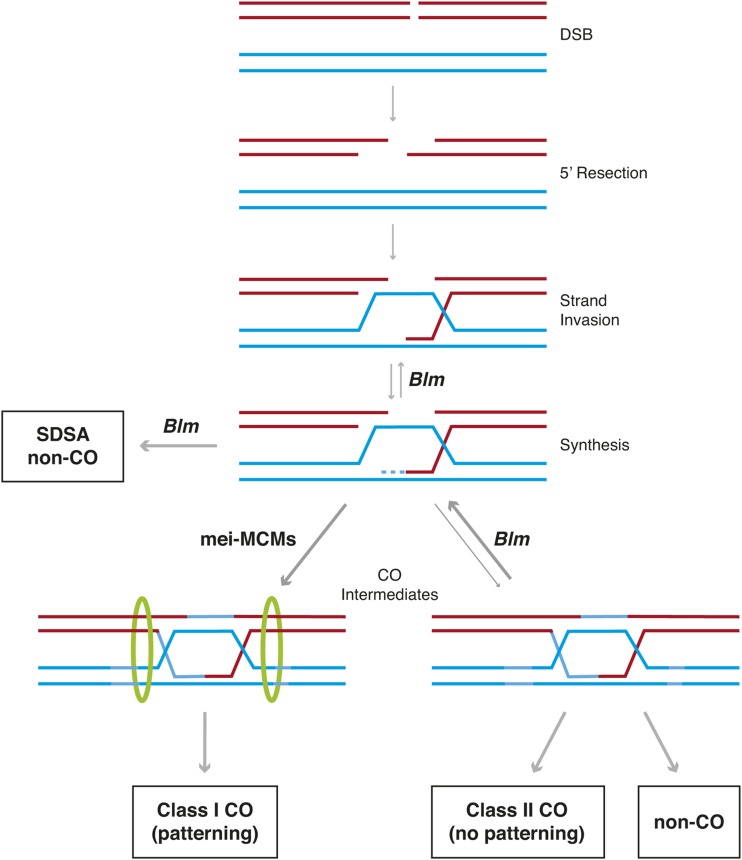
Meiotic recombination in *Drosophila*. During meiotic recombination programmed DSBs are formed, the 5′ ends are recessed and the resulting 3′ single-stranded tails invade the homologous chromosome. After synthesis off the template, the BLM Helicase can unwind the structure which is then resolved via synthesis-dependent strand annealing (SDSA) into a non-crossover gene conversion. If the invading strand synthesizes far enough, second-end capture can occur, creating a crossover intermediate. Most crossover intermediates are processed via the class I pathway in which they are stabilized by the mei-MCM complex (green rings) and resolved into interfering crossovers. A smaller fraction of crossover intermediates enters the class II pathway in which they are resolved as either non-interfering crossovers or non-crossovers with equal probability. The BLM Helicase inhibits crossover intermediate processing though the class II pathway and therefore promotes the class I pathway.

Recently, we showed that the dicistronic gene, *mei-217/mei-218* (hereafter *mei-217/-218*), is a major contributor to evolved species differences in recombination rate and patterning between *D. melanogaster* and *D. mauritiana* (Brand *et al.* 2018). The total genetic map of *D. mauritiana* is ∼1.8-fold longer than that of *D. melanogaster*, and the chromosomal distribution of crossover events differs between species (True *et al.* 1996). This is most evident in telomere- and centromere-proximal regions where crossover formation is more suppressed in *D. melanogaster* than *D. mauritiana* (True *et al.* 1996). When a wildtype *D. mauritiana* allele of *mei-217/-218* is transgenically introduced into mutant *D. melanogaster* females lacking *mei-218* function, a largely (∼82%) *D. mauritiana*-like genetic map is observed (Brand *et al.* 2018). The *D. mauritiana* allele of *mei-217/-218* results in weaker telomeric and centromeric suppression of crossing over as well as reduced crossover interference in medial euchromatic regions (Brand *et al.* 2018). Although *mei-217* and *mei-218* are encoded on a single transcript with two translation start sites that yield two distinct proteins (Liu *et al.* 2000), population genetic signals of recurrent positive selection in the *D. melanogaster* and *D. mauritiana* lineages localize exclusively to *mei-218* (Brand *et al.* 2018). These findings imply that species differences in meiotic crossing over can be mediated by adaptive evolution at *mei-218*.

In this paper, we study the long-term molecular evolution of MEI-218, and its interacting proteins, and we test for further evidence of its functional divergence in other *Drosophila* lineages. First, we ask if recurrent positive selection at *mei-218* is limited to the *D. melanogaster* and *D. mauritiana* lineages or instead extends to the broader *Drosophila* phylogeny, as seen with *Prdm9* in rodents and primates (Oliver *et al.* 2009). We survey protein-coding sequence evolution at genes encoding components of the mei-MCM complex (*mei-218*, *mei-217*, *rec*, *Mcm5*) and the *Blm* helicase among members of the *melanogaster*, *obscura*, and *virilis* species groups. Our analyses reveal evidence for recurrent positive selection at two genes— *mei-218* and *rec*— in the *melanogaster* and in the *obscura* species groups. Second, we ask if evolution at *mei-218* has functional consequences for meiotic crossing over. The genetic maps of *D. pseudoobscura* and *D. virilis* are ∼2 times longer than that of *D. melanogaster* (Ortiz-Barrientos *et al.* 2006; L. Hemmer *et al*. unpublished). While substitutions at *mei-218* might have affected crossing over, it is also possible that they were inconsequential (neutral) or mediated the evolution of some other (unknown) function (*e.g.*, in the male germline; Chintapalli *et al.* 2007; Chen *et al.* 2014). To distinguish these possibilities, we assayed the meiotic crossover phenotypes of transgenes bearing wildtype *D. pseudoobscura* or *D. virilis* alleles in an otherwise *D. melanogaster* genetic background. Our transgene experiments show that both species’ alleles have functionally diverged from that of *D. melanogaster*: for the *D. pseudoobscura* allele, overall crossover rates are comparable to *D. melanogaster* whereas crossover patterning shifts to a more uniform distribution; for the *D. virilis* allele, crossover rates are aberrantly low compared to wildtype whereas crossover patterning is comparable to *D. melanogaster*. These observations suggest that crossover rate and crossover patterning may be separable functions.

## Materials and Methods

### Alignments and PAML analyses

We extracted and aligned the coding sequences of five genes, *mei-217*, *mei-218*, *rec*, *Mcm5*, and *Blm* from 23 species spread across the *melanogaster*, *obscura*, and *virilis* groups (Table S1). Coding sequences for *D. affinis* and the species in the *virilis* group were generously provided by Rob Unckless (University of Kansas) and Yasir Ahmed-Braimah (Cornell University), respectively. Once all coding sequences for each gene were compiled for each of the three species groups, we translated the coding sequence into predicted protein sequences, aligned the amino acid sequences using MUSCLE v3.8.425 (Edgar 2004), then back-translated the alignment into the original nucleotide sequences. CDS alignments were assessed and gap-adjusted by hand to retain in-frame codons (alignments available by request). Each gene alignment was fit to an NSsites model, part of the CODEML package in PAML (Yang 1997). We compared model 7 (M7), which does not allow *d*_N_*/d*_S_ to exceed 1 for any codons, to model 8 (M8) which allows *d*_N_*/d*_S_ > 1 for a subset of codons. We used a likelihood ratio test to determine the best fit model. Sites identified as having experienced positive selection were those found to have posterior probabilities >95% with Bayes Empirical Bayes (Yang *et al.* 2005).

### Generating transgenic flies

To generate transgenic flies, we cloned the *D. pseudoobscura* and *D. virilis* alleles of *mei-217/-218* and used the Φ*C31* integrase to place the transgenes in (the same) desired chromosomal landing sites via site-specific integration (Venken *et al.* 2006). We amplified three sections of the *D. pseudoobscura mei-217/-218* extended gene region with associated 5′ and 3′ noncoding regions from the *D. pseudoobscura* reference genome strain using iProof polymerase (Bio-Rad, Hercules, CA) and sequentially reconstructed the fragments within a pBluescript KS+ vector in a three-step process using standard molecular techniques. The first fragment, containing the upstream region and most of *mei-217*, was amplified as a 2.2kb fragment, phosphorylated, and cloned into the *Spe*I site of KS+ generating an intermediate plasmid KS[pse^frag1^]. The second fragment, containing the 3′ end of *mei-217* and most of *mei-218*, was amplified as a 2.3kb fragment, phosphorylated, and cloned separately into the *Spe*I site of KS+ generating a second intermediate plasmid KS[pse^frag2^]. We then digested KS[pse^frag1^] with *XbaI* and *NotI* (New England Biolabs, Ipswich, MA), gel purified the resulting 2.2kb fragment, and cloned it upstream of KS[pse^frag2^] into *XbaI*/*NotI* sites. This generated a third intermediate plasmid KS[pse^frag1+2^]. The third fragment, containing the 3′ end of *mei-218* and downstream 3′ non-coding regions, was amplified and digested with *XhoI* and *NarI* (New England Biolabs, Ipswich, MA) resulting in a 1.4kb fragment which was subsequently cloned into the *XhoI*/*NarI* sites downstream of KS[pse^frag1+2^] generating the final plasmid KS[pse^frag1+2+3^]. This final recombinant plasmid, KS[pse^frag1+2+3^], reconstitutes the entire *D. pseudoobscura* 5.7kb *mei-217/-218* gene region. We confirmed the absence of introduced mutations in the cloned *mei-217/-218*^pse^ allele by direct Sanger sequencing of the KS[pse^frag1+2+3^] plasmid. We then cut the *mei-217/-218* insert from the KS+ vector with *NotI* and subcloned into an *attB*[Pacman]-Ap^R^ vector obtained from the *Drosophila* Genomics Resource Center (Bloomington, IN).

In an analogous manner, the *D. virilis mei-217/-218* extended gene region was amplified from the *D. virilis* reference genome strain and sequentially reconstructed within a pBluescript KS+ vector (hereafter KS+; Stratagene, La Jolla, CA) in a three-step process using standard molecular biology techniques. All bacterial transformations were performed at room temperature to enhance plasmid stability in One Shot TOP10 chemically competent *E. coli* (Invitrogen, Carlsbad, CA). First, the upstream region and most of *mei-217* was amplified as a 2.6 kb fragment, using a 5′ primer that contained a *SphI* site. The 5′ end of the resulting PCR product was digested with *SphI* (New England Biolabs, Ipswich, MA) while the 3′ end was made blunt using FastAp Thermosenstive Alkaline Phosphatase (Thermo Fisher, Waltham, MA). This fragment was cloned into the *SphI*/*SpeI* sites of KS+, generating intermediate plasmid KS[vir^frag1^]. Second, the 3′ end of the *mei-217* and most of *mei-218* was amplified as a 3.2kb fragment. The amplicon was phosphorylated with T4 Polynucleotide Kinase (Invitrogen, Carlsbad, CA) and ligated into the *Spe*I site of KS+ generating a second intermediate plasmid KS[vir^frag2^]. We then digested KS[vir^frag2^] with *AatII* and *NotI* (New England Biolabs, Ipswich, MA), gel purified the resulting 3.2kb fragment, and cloned it into the *SphI*/*SmaI* sites downstream of the vir^frag1^ generating a third intermediate plasmid KS[vir^frag1+2^]. Third, the 3′ end of *mei-218* and downstream 3′ non-coding regions was amplified and digested with *AatII* and *SalI* (New England Biolabs, Ipswich, MA) resulting in a 1.1kb fragment which was subsequently cloned into the *AatII*/*SalI* sites downstream of KS[vir^frag1+2^] generating the final plasmid KS[vir^frag1+2+3^]. This final recombinant plasmid, KS[vir^frag1+2+3^], reconstitutes the entire *D. virilis* 6.9kb *mei-217/-218* gene region. We then cut the *mei-217/-218* insert from the KS+ vector with *NotI* and subcloned into an *attB*[Pacman]-Ap^R^ vector obtained from the *Drosophila* Genomics Resource Center (Bloomington, IN). We confirmed the absence of introduced mutations in the cloned *mei-217/-218*^vir^ allele by Sanger sequencing.

Both *D. pseudoobscura* and *D. virilis* transgene constructs were introduced into *D. melanogaster y w*; *PBac*[*y*^+^-attP-9A]VK00005 flies, which have an *attP* transgene landing site at cytological position 75A10 on chromosome arm *3L*, via injections performed by BestGene (Chino Hills, CA). The *attB-P*[*w*^+^ *mei-218*^pse^]-Ap^R^ and *attB-P*[*w*^+^ *mei-218*^vir^]-Ap^R^ transgenic flies (for simplicity, hereafter referred to as *P*[*mei-217/218*^pse^] and *P*[*mei-217/-218*^vir^], respectively) were then made homozygous and maintained as stocks. Following the crossing protocol in (Brand *et al.* 2018), we estimated crossover rates for a multiply marked second chromosome in two female genotypes:

1. *mei-218*^1^; *net ho dp b pr cn*/ + + + + + +; *P*[*mei-217*/-*218*^pse^]/ +; and
2. *mei-218*^1^; *net ho dp b pr cn*/ + + + + + +; *P*[*mei-217*/-*218*^vir^]/ +.

To estimate crossover frequencies, we crossed the female genotypes above to homozygous *net dpp*^*d-ho*^ *dp b pr cn* males and scored the progeny for all markers. (For clarity, we refer to *dpp*^*d-ho*^ throughout by its mutant synonym, *ho*.) We performed *n* = 13 and *n* = 14 crosses for *mei-217*/-*218*^pse^ and *mei-217*/-*218*^vir^, respectively (see Table S2 for data). For each cross, we collected either ∼10 virgin *mei-218*^1^; *net ho dp b pr cn/ + + + + + +*; *P*[*mei-217/-218*^pse^]/+ females or *mei-218*^1^; *net ho dp b p cn/ + + + + + +*; *P*[*mei-217/-218*^vir^]/+ females, aged them for three to five days, and crossed them to ∼10 *net ho dp b pr cn* males that were aged for at least two days. After five days, parents were discarded, and the vials were hydrated with a solution of 0.5% propionic acid. All crosses were maintained in an incubator at 24C under a 12-hour light/dark cycle on standard corn-meal media. We estimated means and standard deviations of crossover frequency among the independent, replicate crosses and compared genotypes using standard *t*-tests (Figure 3B,4B).

To compare the spatial distribution of crossovers across the five intervals spanning the *net-cn* region (Table S3) while controlling for overall crossover rate differences, we compared standardized distributions among genotypes based on the proportion of crossovers occurring in each interval using *χ*^2^ tests. We also tested for genotypic differences in crossover event distributions among tetrads by inferring the frequencies of non-, single-, double- or triple crossovers (*E*_0_, *E*_1_, *E*_2_, and *E*_3_, respectively) using the algebraic methods of Weinstein (1936). We estimated the strength of Interference (*I*) as 1− (observed double crossovers / expected double crossovers). All statistical analyses were performed using *R* (http://www.R-project.org/). The *Drosophila* stocks and plasmids used in this study are available upon request. The authors affirm that all data necessary for confirming the conclusions of the article are present within the article, figures, and tables.

### Data Availability

Fly stocks and transgenic constructs are available upon request. The authors affirm that all data necessary for confirming the conclusions of the article are present within the article, figures, and tables. Supplemental material available at FigShare: https://doi.org/10.25387/g3.9162431.

## Results

### Positive selection at meiosis genes that regulate crossover formation and patterning

**Figure 2:**
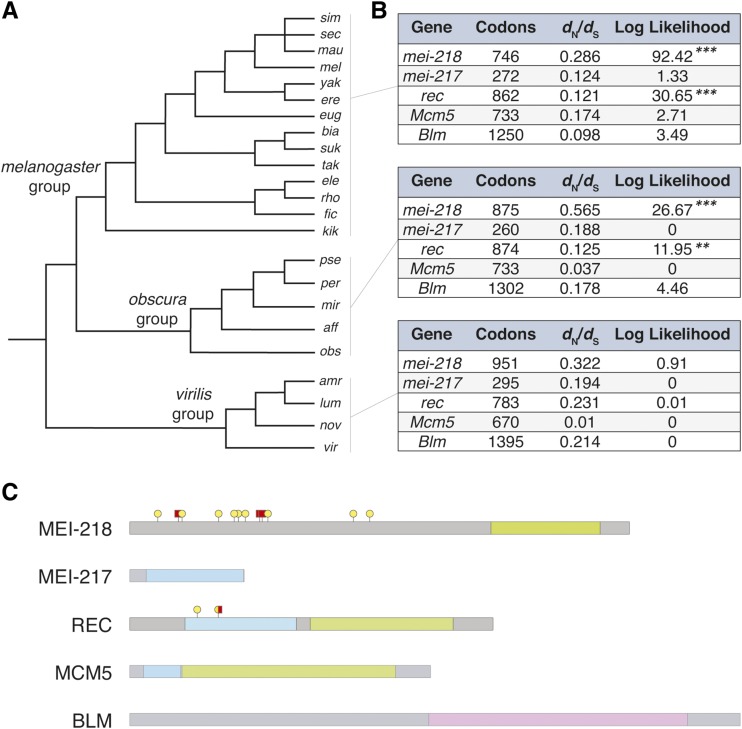
Molecular evolution across the *Drosophila* phylogeny. (A) Phylogenetic relationship of the 23 species analyzed within the *melanogaster*, *obscura*, and *virilis* species group. (B) PAML analyses for the three species groups were performed separately because the phylogenetic distances among them is so large that *d*_s_ is saturated. We report the log-likelihood estimates from a model 7 – model 8 comparison (*P < 0.05, **P < 0.01, ***P < 0.001). (C) A schematic of the structural domains in the five proteins analyzed. In the MEI-MCMs the AAA ATPase MCM domains are shaded in green and the MCM N-terminal domains are shaded in blue. In the BLM Helicase the RecQ DNA Helicase domain in shaded in purple. In MEI-218 and REC, the pins represent codons with evidence for positive selection in the *melanogaster* group (yellow circle) and the *obscura* group (red squares).

We identified and extracted protein-coding sequences encoding members of the mei-MCM complex (*mei-218*, *mei-217*, *rec*, *Mcm5*) and, because it antagonizes the mei-MCMs, *Blm* (Kohl *et al.* 2012), from whole genome sequence data of 23 species (see Materials and Methods, Table S1). In some cases, we used Sanger sequencing to complement retrieved sequence data that had gaps and/or quality issues (Table S1). To investigate patterns of long-term molecular evolution, we used maximum likelihood methods to test for phylogenetic evidence of positive selection. We analyzed the *melanogaster* (*n* = 14), *pseudoobscura* (*n* = 5), and *virilis* (*n* = 4) species groups separately, as the phylogenetic distances among the three groups are so large that synonymous site divergence (*d*_S_) is saturated (Figure 2A; Larracuente *et al.* 2008; Stanley and Kulathinal 2016). Using the codeml program in the PAML suite, we performed likelihood ratio tests to identify genes that have elevated rates of nonsynonymous substitution relative to synonymous substitution (*d*_N_*/d*_S_) (Yang 1997). In particular, we compared the log-likelihood of a model for which the estimated values of *d*_N_*/d*_S_ for individual codons is *β*-distributed between 0 and 1 (model 7) to that of an alternative model for which the distribution includes an additional class of codons with *d*_N_*/d*_S_ > 1 (model 8). Positive selection is inferred for cases in which model 8 provides a significantly better fit to the data (Yang 1997; see Materials and Methods).

Two of the five genes show phylogenetic evidence of recurrent positive selection. First, we find that *mei-218* has a history of positive selection in both the *melanogaster* and *obscura* species groups (Figure 2B). A Bayes Empirical Bayes (BEB) analysis (Yang *et al.* 2005) identified nine codons in the *melanogaster* group and three codons in the *obscura* group, all clustered in the second and third exons, with evidence of positive selection (Figure 2C; posterior probability >0.95). The quality of the local sequence alignment is low for both species groups, compromising our confidence in the BEB-identified codons. To address this problem, we performed PAML analyses on each of the five exons, separately, to broadly localize the signal of positive selection within *mei-218*. Consistent with the codons identified by the BEB analysis, we find that the second and third exons, which encode a disordered protein region, show evidence of positive selection in both the *melanogaster* and the *obscura* species groups. We aligned coding sequences using MUSCLE (Edgar 2004), although other alignment algorithms (*i.e.*, ClustalW and Geneious) give qualitatively similar results. The alignment uncertainty results partly from high rates of indel evolution at *mei-218*. For example, compared to the *D. melanogaster* reference, sequences from the other species of the *melanogaster* group contain ≥13 indels ranging in size from 1 to 267 codons. This frequent insertion and/or deletion of codons limits the power of our PAML analysis of *mei-218*: of the 5,166 sites in the *mei-218 melanogaster* group alignment, 2,967 sites (57%) were not analyzed due to gaps in the multi-species alignment.

Among the other four genes studied, only *rec* shows evidence of positive selection in the *melanogaster* and *obscura* species groups (Figure 2B). BEB analyses identified two codons in the MCM N-terminal domain with histories of positive selection (Figure 2C; posterior probability >0.95): one codon experienced positive selection in the *melanogaster* group and the other codon experienced positive selection in both the *melanogaster* and *obscura* groups. At the positively selected codon detected in the *melanogaster* group, eight different amino acid states are represented among 14 species. At the positively selected codon detected in both the *melanogaster* and *obscura* groups, different amino acid states are represented in 8/14 and 4/5 species, respectively. PAML analyses find no support for positive selection at the remaining three genes (*mei-217*, *Mcm5*, or *Blm*). In the *virilis* species group, none of the five genes tested show evidence for positive selection (Figure 2B).

### Functional analysis of mei-217/-218 from D. pseudoobscura

*D. pseudoobscura* diverged from *D. melanogaster* ∼30 mya, and its genetic map is ∼2-fold longer, with less expansive centromeric suppression of crossing over and a more uniform recombination landscape (Hamblin and Aquadro 1999; Ortiz-Barrientos *et al.* 2006; Drosophila 12 Genomes Consortium 2007). The levels of coding sequence and length divergence between *D. pseudoobscura* and *D. melanogaster* are extraordinary, and different regions of the *mei-217/-218* sequence have experienced strikingly different rates of molecular evolution. Between *D. pseudoobscura* and *D. melanogaster*, pairwise amino acid identity for *MEI-218* is much lower for than for *MEI-217* (∼34% *vs.* 61%, respectively). The very low identity for *MEI-218* is attributable to the N-terminal disordered region— which shares only ∼20% identity and differs in length by 183 codons— not the C-terminal AAA ATPase MCM domain which shares 70% identity (Figure 2C). Given the extraordinary protein sequence (and indel) divergence at *MEI-218*, the statistical evidence for recurrent positive selection at *mei-218* in both species groups, and previous experimental evidence that *mei-217/-218* mediates species differences in crossing over (Brand *et al.* 2018), we sought to test if molecular evolution at *mei-217/-218* between *D. pseudoobscura* and *D. melanogaster* has functional consequences for crossing over.

**Figure 3:**
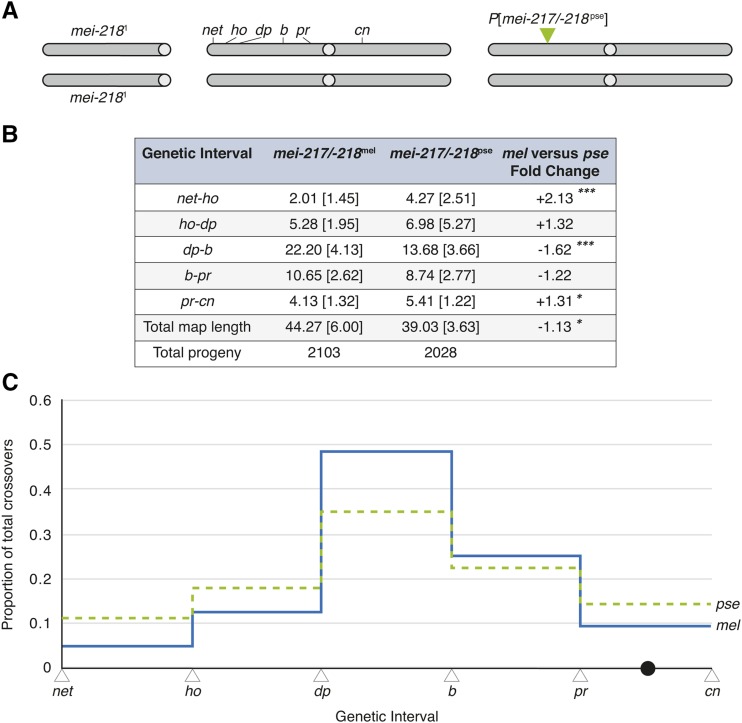
The *mei-217/-218* allele of *D. pseudoobscura* alters the rate and patterning of crossing over in *D. melanogaster*. (A) *D. melanogaster* females containing a transgene of a *D. pseudoobscura mei-217/-218* allele inserted on chromosome *3L* (75A10) were assayed for crossing over. The endogenous *mei-218*^1^ allele contains a nonsense mutation. Crossover frequencies were estimated among the six visible markers spanning the left arm of chromosome *2* and the centromere: *net* (*net*), *decapentaplegic* (*ho*), *dumpy* (*dp*), *black* (*b*), *purple* (*pr*), and *cinnabar* (*cn*). *mei-217/-218*^mel^ data re-produced from Brand *et al.* (2018). (B) For each genotype, the means and standard deviations [in brackets] of crossover frequency for the five genetic intervals measured in the two transgenic genotypes. The *p*-values are for unpaired *t*-tests (*P < 0.05, **P < 0.01, ***P < 0.001). (C) The proportion of total crossovers distributed across the five intervals in the *net-cn* region in *mei-217/-218*^mel^ (blue) and *mei-217/-218*^pse^ (green) females. The total number of crossovers scored for *mei-217/-218*^mel^ and *mei-217/-218*^pse^ females is 956 and 786, respectively (see Table S2).

To experimentally test for functional effects of molecular divergence at *mei-217/-218* between *D. pseudoobscura* and *D. melanogaster*, we cloned the entire *mei-217/-218* gene region, including all of the upstream and downstream noncoding regions, from *D. pseudoobscura* into an *attB-P*[acman] vector (hereafter *mei-217/-218*^pse^; see Materials and Methods). We integrated this transgene construct into an *attP* site on chromosome arm *3L* of *D. melanogaster* (cytological position 75A10) and used genetic crosses to place the transgene in a *mei-218* loss-of-function genetic background resulting in the *D. melanogaster* stock, *mei-218*^1^; *P*[*mei-217/-218*^pse^] (see Materials and Methods). We then estimated crossover frequencies among six visible markers (*net ho dp b pr cn*) that span chromosome arm *2L* and the centromere for replicate crosses of *mei-218*^1^; *net ho dp b pr cn*/+ + + + + +; *P*[*mei-217/-218*^pse^]/+ females to *net ho dp b pr cn* males (*n* = 13 crosses, 2028 progeny; Figure 3, Table S2). In this genotype, the *D. pseudoobscura* wildtype allele is the only source of *mei-218* function. As our transgenes include *mei-217/-218* coding and non-coding sequence, we are unable to attribute phenotypic effects to species differences in the protein sequence *vs.* expression level.

These experiments have three possible outcomes. First, sequence evolution at *mei-217/-218* may be of no functional consequence to crossing over: *mei-217/-218*^pse^ might rescue the *mei-218* mutant phenotype but produce wildtype *D. melanogaster*-like rates and patterning of crossing over. Second, sequence evolution at *mei-217/-218* may render it incompatible between species: the *mei-217/-218*^pse^ allele might be sub- or non-functional in *D. melanogaster* so that it cannot fully rescue the *mei-218* mutant phenotype. Last, sequence evolution at *mei-217/-218* may recapitulate some of the wildtype species differences in crossing over: the *mei-217/-218*^pse^ allele might rescue the null *mei-218* mutant phenotype but produce rates and/or patterning of crossing over that differ from *D. melanogaster* in a way similar to *D. pseudoobscura*.

We find that, in a *D. melanogaster* genetic background lacking *mei-218* function, the *mei-217/-218*^pse^ rescues crossing over: the total *net-cn* genetic map length of 39.03 map units is smaller (∼1.13-fold) than, but comparable to *mei-217/-218*^mel^ controls (*t*-test, *P* = 0.044; Figure 3B). However, while the total lengths of the *mei-217/-218*^pse^ and *mei-217/-218*^mel^ genetic maps are comparable, we observe highly significant crossover rate heterogeneity among intervals between the transgenes. In *mei-217/-218*^pse^ females, the medial largest interval of chromosome arm *2L* (*dp-b*) experiences a 1.6-fold lower crossover frequency than the *mei-217/-218*^mel^ control (*t*-test, *P* < 0.0001; Figure 3B) whereas the telomere-proximal (*net-ho*) and centromere (*pr-cn*) regions experience 2.13- and 1.31-fold higher crossover frequencies, respectively (*t*-test, *P* = 0.001 and *P* = 0.028, respectively; Figure 3B). To distinguish crossover frequency and crossover patterning, we calculated the proportion of total crossovers that occurred in each genetic interval for each transgene. These values differ from genetic map distances (the proportion of recombinant progeny), for which crossover rate and patterning are confounded, and instead provide profiles of crossover patterning that are independent of crossover rate. We find that the patterning of crossovers differs significantly between *mei-217/-218*^mel^ and *mei-217/-218*^pse^ (*χ*^2^ test, *df* = 4, *P* < 9.52e^-28^; Figure 3C, Table S3). Specifically, crossovers in *mei-217/-218*^mel^ females are concentrated in the medial *ho-dp-b* regions and occur at lower frequencies in the telomeric *net-ho* and centromeric *pr-cn* regions, resulting in relatively larger variability in the proportion of crossovers among the genetic intervals (range between lowest *net-ho* and highest *dp-b* intervals = 0.44; Figure 3B,C; Table S3). In contrast, crossovers in *mei-217/-218*^pse^ females are distributed more uniformly across the genetic intervals, with less variability in the proportion of crossovers among the genetic intervals (range between lowest *net-ho* and highest *dp-b* intervals = 0.24; Figure 3B,C; Table S3). The *mei-217/-218*^pse^ transgene thus produces a total genetic map length that is nearly *D. melanogaster*-like but crossover patterning that differs from *D. melanogaster* in ways similar to *D. pseudoobscura*.

The distribution of the number of crossovers per recovered chromosome— the number of non-crossover (NCO), single-crossover (SCO), double-crossover (DCO) chromosomes, and so on— differs significantly between *mei-217/-218*^pse^ and *mei-217/-218*^mel^ transgenes (*χ*^2^ test, *df* = 3, *P* < 1.29e^-16^; Table S2). We used these data to infer the distribution of the number of crossovers per tetrad using the methods of Weinstein (1936). Two mechanisms constrain the distribution of the number of crossovers per meiosis: (1) crossover assurance encourages the formation of at least one obligate crossover per tetrad to guarantee proper segregation; and (2) crossover interference discourages the formation of multiple crossovers near one another (Jones and Franklin 2006; Berchowitz and Copenhaver 2010; Wang *et al.* 2015). As a result, the number of crossovers per tetrad is under-dispersed relative to Poisson expectations, with zero- and multiple-crossover classes under-represented in wildtype *D. melanogaster* (Mehrotra *et al.* 2007). Consistent with regulation, the inferred number of crossovers per tetrad is similarly under-dispersed relative to Poisson expectation in *mei-217/-218*^pse^ females (*χ*^2^ test, *df* = 5, *P* < 1.3e^−237^). However, the distribution of the number of crossovers per tetrad differs between the *mei-217/-218*^pse^ and *mei-217/-218*^mel^ females (*χ*^2^ test, *df* = 3, *P* = 2.2e^−16^; Table 1). The mean number of crossovers per tetrad is reduced in *mei-217/-218*^pse^ females compared to *mei-217/-218*^mel^ controls (0.78 *vs.* 0.91). This difference is largely attributable to a 1.9-fold increase in non-crossover (*E*_0_) tetrads at the expense of single-crossovers (*E*_1_), which are reduced 1.47-fold (Table 1). Crossover assurance thus appears weaker in *mei-217/-218*^pse^ females. At the same time, however, we find that multiple-crossover tetrads are more frequent in *mei-217/-218*^pse^ females: double- and triple-crossover tetrads are increased 1.16- and 5-fold, respectively (Table 1). We tested whether this increase in multi-crossover tetrads is enabled by weaker crossover interference in *mei-217/-218*^pse^ females. Indeed, crossover interference for the two largest adjacent intervals (*ho-b-pr*) is ∼28% weaker in *mei-217/-218*^pse^ females, although the difference is not significant (*I* = 0.567 *vs.* 0.793, Mann-Whitney, *P* = 0.795; Table S2). Together, the weaker crossover assurance and crossover interference in *mei-217/-218*^pse^ females increase the relative variance (variance/mean) in the number of crossovers per tetrad (0.698) compared to that for in *mei-217/-218*^mel^ (0.350; Table 1).

**Table 1:** The distribution of the inferred number of crossovers in the net-cn region per meiosis differs among genotypes

Tetrad Class#	*mei-218*^1^‡	*mel*‡	*pse*	*vir*	*pse-mel* fold-diff*	*vir-mel* fold-diff*	*vir-pse* fold-diff*
*E*_0_	0.958	0.205	0.389	0.885	+1.90	+4.32	+2.27
*E*_1_	0.042	0.685	0.467	0.094	−1.47	−7.29	−4.97
*E*_2_	0.000	0.107	0.124	0.015	+1.16	−7.13	−8.27
*E*_3_	0.000	0.004	0.020	0.006	+5	+1.5	−3.33
Mean†	0.042	0.909	0.775	0.143			
Variance†	0.000	0.319	0.541	0.190			
Relative variance^	0.000	0.350	0.698	1.33			

# *E*_0,_ *E*_1,_ *E*_2,_ *E*_3_ are the estimated frequencies of tetrads with zero, one, two and three inferred crossovers, respectively. Tetrad frequencies were estimated using Weinstein's (1936) algebraic method.

‡ *mei-218*^1^, *mei-217/-218*^mel^ data are reproduced from Brand *et al*. (2018).

* Weinstein estimates for *mei-217/-218*^mel^, *mei-217/-218*^pse^, and *mei-217/-218*^vir^ alleles differ significantly from one another (*χ*^2^ test, *df* = 5, *P* < 1.49e^-130^).

† Mean and variance = mean and sample variance of the inferred number of crossovers per tetrad, respectively.

^ Relative variance = variance/mean.

These results show that crossover assurance, interference, and centromeric and telomeric suppression are all weaker in *mei-217/-218*^pse^ females. These observations are qualitatively consistent with crossover patterning in wildtype *D. pseudoobscura*, which also shows reduced (or even possibly absent) centromeric suppression and a more uniform distribution of crossovers (Hamblin and Aquadro 1999; Ortiz-Barrientos *et al.* 2006; Kulathinal *et al.* 2008). Alternatively, it is possible that the *mei-217/-218*^pse^ allele is incompatible with interactors from *D. melanogaster*, so that the functionally divergent *mei-217/-218*^pse^ allele is unable to fully receive and/or implement endogenous crossover patterning signals from *D. melanogaster*. Under this incompatibility hypothesis, the shift in crossover patterning toward one that is *D. pseudoobscura*-like would be coincidental rather than a reflection of the wildtype properties of the *D. pseudoobscura* allele. As with any heterologous transgene (or interspecific genetic) experiment, formally distinguishing between these two interpretations is difficult. However, under either interpretation, the results demonstrate that the effects of *D. pseudoobscura* and *D. melanogaster* alleles on meiotic crossing over have diverged and support the notion that *mei-217/-218* has two separable functions— crossover formation and crossover patterning.

### Functional analysis of mei-217/-218 From D. virilis

We also assayed a *mei-217/-218* wildtype allele from a species more distantly related to *D. melanogaster*. *D. virilis* diverged from *D. melanogaster* ∼50 mya and has a ∼2-fold longer genetic map (L. Hemmer *et al*. unpublished; Drosophila 12 Genomes Consortium 2007). Although our PAML analyses failed to detect evidence of positive selection at *mei-218* within the *virilis* species group, these analyses are uninformative about the possibility of positive selection in the lineages ancestral to the *virilis* and *melanogaster* species groups. The *MEI-217* protein shows ∼58% pairwise amino acid sequence identity between *D. melanogaster* and *D. virilis*. In contrast, the *MEI-218* protein shares only ∼34% pairwise amino acid sequence identity overall. Divergence at *mei-218* is not uniform across the protein: while the C-terminal AAA ATPase MCM domain shows ∼63% identity, the disordered N-terminal shows just ∼23% identity (Figure 2C; see also Manheim *et al.* 2002). The *D. virilis* protein is 954 amino acids long, shorter than both *D. melanogaster* (1186 aa) and *D. pseudoobscura* (1002 aa). The difference in length is driven by indel evolution largely concentrated in the disordered region of *MEI-218*, as the N-terminal MCM domain has remained relatively unchanged between the three species (*D. melanogaster* 337 aa; *D. pseudoobscura* 336 aa; *D. virilis* 334 aa; see also Kohl *et al.* 2012).

**Figure 4:**
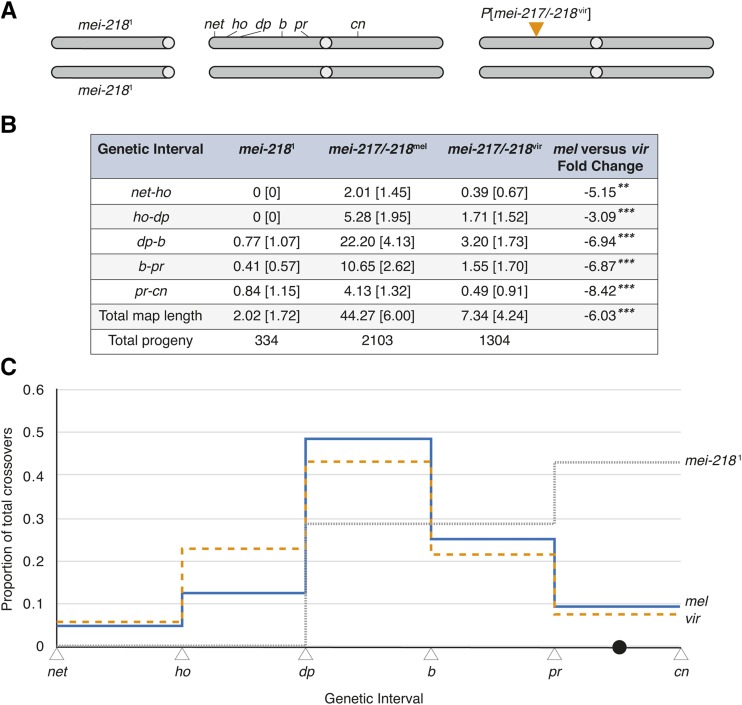
The *mei-217/-218* allele of *D. virilis* alters the rate and patterning of crossing over in *D. melanogaster*. (A) *D. melanogaster* females containing a transgene of a *D. virilis mei-217/-218* allele inserted on chromosome *3L* (75A10) were assayed for crossing over. The endogenous *mei-218*^1^ allele contains a nonsense mutation. Crossover frequencies were estimated among the six visible markers spanning the left arm of chromosome *2* and the centromere: *net* (*net*), *decapentaplegic* (*ho*), *dumpy* (*dp*), *black* (*b*), *purple* (*pr*), and *cinnabar* (*cn*). *mei-218*^1^ and *mei-217/-218*^mel^ data re-produced from Brand *et al.* (2018). (B) For each genotype, the means and standard deviations [in brackets] of crossover frequency for the five genetic intervals measured in the *mei-218* mutant and two transgenic genotypes. *p*-values are derived for unpaired *t*-tests. *P < 0.05, **P < 0.01, ***P < 0.001. (C) The proportion of total crossovers distributed across the five intervals in the *net-cn* region in *mei-217/-218*^mel^ (blue), *mei-217/-218*^vir^ (orange), and *mei-218*^1^ (gray) females. The total number of crossovers scored for the *mei-217/-218*^mel^, *mei-217/-218*^vir^, and *mei-218*^1^ females is 956, 93, and 7, respectively (see Table S2).

To functionally assay the *D. virilis* allele of *mei-217/-218*, we followed the same strategy used to create transgenic flies with *mei-217/-218*^pse^ (see above) and *mei-217/-218*^mau^ (Brand *et al.* 2018). We first cloned the entire *D. virilis mei-217/-218* gene region and flanking non-coding sequences into an *attB-P*[acman] vector (hereafter *mei-217/-218*^vir^) and integrated it into an *attP* site on *3L* (75A10) in *D. melanogaster* (see Materials and Methods). We then used crosses to place the transgene into a *D. melanogaster mei-218* mutant background and measured crossover frequencies among visible markers spanning part of the second chromosome in replicate *mei-218*^1^; *net ho dp b pr cn*/+ + + + + +; *P*[*mei-217/-218*^vir^]/+ females (*n* = 14 crosses, 1304 progeny; Figure 4, Table S2; see Materials and Methods).

Rates of crossing over are strongly reduced in *mei-217/-218*^vir^ transgene-bearing females (Figure 4B). While the control *mei-217/-218*^mel^ transgene produces a total genetic map length of 44.27 across the *net-cn* region, the *mei-217/-218*^vir^ transgene produces a genetic map that is ∼6-fold smaller (7.34; *t*-test, *P* < 0.0001; Figure 4B). Two lines of evidence, however, indicate that *mei-217/-218*^vir^ does not behave like a null allele in *D. melanogaster*: compared to *mei-218*^1^ mutant females, *mei-217/-218*^vir^ females produce longer genetic maps (2.02 map units; *t*-test, *P* = 0.001; Figure 4B) and show non-uniform spatial patterning of crossovers. In mutant *mei-218*^1^ females, ∼90% of crossovers are eliminated, and the residual crossovers fail to show crossover interference or centromeric suppression (Baker and Carpenter 1972); see also Figure 4C, Table S3). These observations suggest that most crossovers in wildtype females correspond to interfering class I crossovers, whereas residual crossovers in *mei-218*^1^ females correspond to non-interfering class II crossovers which originate via a different pathway (Figure 1; Berchowitz and Copenhaver 2010; Kohl and Sekelsky 2013). To determine if the crossovers in *mei-217/-218*^vir^ behave like residual (presumed class II) crossovers of *mei-218*^1^ females, we tested whether crossover patterning across the genetic intervals is disrupted. The distribution of crossovers between *mei-217/-218*^vir^ and *mei-218*^1^ is significantly different (*χ*^2^ test, *df* = 4, *P* < 1.2e^-12^; Figure 4C, Table S3). Specifically, *mei-218*^1^ females show a non-uniform reduction in crossover frequency, with crossing over reduced in centromere-proximal regions (*pr-cn*) to only 20.3% of the *mei-217/-218*^mel^ value compared to 4.6% across the entire *2L* (*net-cn*) region (see also Carpenter and Sandler 1974). In contrast, *mei-217/-218*^vir^ females show comparable reductions in crossover frequency among regions, with crossing over reduced in the centromere-proximal regions (*pr-cn*) to 11.9% of the *mei-217/-218*^mel^ value compared to 16.6% across the entire *2L* (*net-cn*) region (Figure 4B). As a result, crossover patterning in *mei-217/-218*^vir^ females is comparable to *mei-217/-218*^mel^ (*χ*^2^ test, *df* = 4, *P* = 0.05; Figure 4C). In *mei-217/-218*^vir^ females, then, overall crossover frequencies are reduced 83.4%, but gross crossover patterning is largely unchanged. These findings show that *mei-217/-218*^vir^ is unable to support wildtype (*D. melanogaster* or *D. virilis*) rates of crossing over in a *D. melanogaster* genetic background but appears able to integrate patterning information specified by *D. melanogaster*.

The distributions of the observed number of crossover events per recovered chromosome (χ^2^ test, *df* = 3 *P* = 7.07e^-137^; Table S2) and the estimated number of crossovers per tetrad helps to explain why the total *net-cn* map length is so much smaller in *mei-217/-218*^vir^ females than *mei-217/-218*^mel^ females. The estimated number of crossovers per tetrad is under-dispersed in females bearing the *mei-217/-218*^mel^ transgene (variance/mean = 0.350), which experience a mean of 0.91 crossovers per tetrad (*χ*^2^ test, *df* = 5, *P* < e^-200^; Table 1; Brand *et al.* 2018). In contrast, the estimated number of crossovers per tetrad is *over*-dispersed in females bearing the *mei-217/-218*^vir^ transgene (variance/mean = 1.33)— with a deficit of single-crossover tetrads (*E*_1_ = 0.094) and an excess of multiple-crossover tetrads (*E*_≥2_ = 0.021; *χ*^2^ test, *df* = 5, *P* = e^-20^; Table 1)— which experience a mean of only 0.14 crossovers per tetrad. The reduced genetic map in *mei-217/-218*^vir^ females therefore occurs because most tetrads experience no crossovers during meiosis. Crossover assurance therefore appears strongly compromised in *mei-217/-218*^vir^ females implying that the *mei-217/-218*^vir^ allele is unable to ensure an obligate crossover in a *D. melanogaster* genetic background. Such achiasmate tetrads suffer elevated rates of mis-segregation and nondisjunction leading to production of aneuploid gametes and reduced fecundity (Baker and Carpenter 1972; Bhagat *et al.* 2004). Consistent with nondisjunction, *mei-217/-218*^vir^ females produce significantly fewer progeny (mean ± SD = 93.14 ± 17.3) than *mei-217/-218*^mel^ females (161.77 ± 52.76; Figure 4B; *t*-test *P* = 0.0005).

The *mei-217/-218*^vir^ allele provides additional evidence that crossover rate and patterning are separable: in *D. melanogaster*, *mei-217/-218*^vir^ is hypomorphic with respect to crossover formation but not crossover patterning. The fact that *mei-217/-218*^vir^ cannot fully complement the *mei-218*^1^-mediated loss of crossover formation in *D. melanogaster* suggests either of two possibilities. The wildtype function of *mei-217/-218*^vir^ may differ from *mei-217/-218*^mel^: whereas *mei-217/-218*^mel^ functions in crossover formation and patterning in *D. melanogaster* (Baker and Carpenter 1972; McKim *et al.* 1996; Kohl *et al.* 2012), *mei-217/-218*^vir^ may be less essential to crossover formation but still essential to crossover patterning in *D. virilis*. Alternatively, *mei-217/-218*^vir^ may be genetically incompatible with factors from *D. melanogaster*: *mei-217/-218*^vir^ may fail to interact appropriately with *D. melanogaster*-encoded proteins such that crossover formation (but not patterning) is compromised. Under either model, the molecular divergence at *mei-217/-218* between *D. melanogaster* and *D. virilis* has functional consequences for female meiosis.

## Discussion

Our phylogenetic analyses revealed that two mei-MCM genes, *mei-218* and *rec*, have histories of recurrent positive selection in the *melanogaster* and *obscura* species groups, and our transgenic assays show that the different species’ *mei-217/-218* alleles have functionally diverged with respect to crossover patterning (*mei-217/-218*^pse^) and crossover formation (*mei-217/-218*^vir^). These observations are superficially reminiscent of the recurrent positive selection at *Prdm9*, the major *trans*-acting factor that controls the distribution of recombination hotspots in mammals. The forces driving the rapid molecular evolution of *Prdm9* are reasonably well understood. During recombination-repair of DSBs, the DNA sequence motifs recognized by the PRDM9 zinc fingers tend to be replaced with non-motif sequence. As the number of recombination hotspots erodes over time, the overall frequency of recombination decreases to suboptimal levels, elevating the risk of chromosomal mis-segregation and/or breakage (Ségurel *et al.* 2011; Smagulova *et al.* 2016). This model explains why there is selection for PRDM9 to acquire novel zinc fingers that recognize novel DNA sequence motifs, creating a new class of recombination hotspots, and thereby reestablishing appropriate recombination frequencies. This process can quickly lead to differences in the identity and distribution of recombination hotspots between closely related species and, incidentally, to sterility in species hybrids (Ptak *et al.* 2005; Davies *et al.* 2016; Smagulova *et al.* 2016). For the mei-MCMs, *mei-218* and *rec*, the causes of recurrent positive selection are unclear. In *Drosophila*, fine-scale heterogeneity in recombination rates exists, but recombination hotspots comparable to those in mammals do not (Comeron *et al.* 2012; Hunter *et al.* 2016). Moreover, unlike PRDM9, the mei-MCMs do not have DNA binding domains known to recognize specific motifs. It therefore seems doubtful that the positive selection we have observed involves DNA motif turnover.

The phylogenetic evidence for recurrent bouts of positive selection at *mei-218* and *rec* are similarly consistent with adaptation to moving fitness optima but the causes of selection are unclear. For instance, despite the absence of crossing over in *Drosophila* males, FlyAtlas and modEncode data show that both *mei-218* and *rec* are expressed in *D. melanogaster* testes (Chintapalli *et al.* 2007; Chen *et al.* 2014). It is therefore possible that the history of positive selection at these genes reflects adaptation for male reproductive functions, although the functions of *mei-218* and *rec* in testes are unknown and mutant males are fertile. If, instead, recurrent positive selection at *mei-218* and *rec* has occurred to modulate crossing over, then we require a model in which the optimal rate and/or distribution of crossing over has changed repeatedly. Selfish genetic elements could provide one source of such fluctuating selection. First, meiotic drive in the female germline can generate selection for modifiers of crossing over. Depending on such details as whether drive occurs in meiosis I or II, or whether drive involves the centromere or telomere(s), selection can favor modifiers that increase or decrease rates of crossing over (Brandvain and Coop 2012). Second, while crossing over provides important meiotic and evolutionary functions, it also entails the risk of ectopic non-homologous exchange between similar but dispersed sequences, like transposons. Ectopic exchange can generate deleterious duplications, deletions, and other chromosomal aberrations (Goldberg *et al.* 1983; Barrón *et al.* 2014). The optimal recombination rate should thus evolve to balance the benefits of crossing over against the costs. The requirement for at least one crossover per chromosome (arm) sets a minimum rate, whereas the risk of ectopic exchange may constrain the maximum rate. The risk of ectopic exchange depends on the abundance of dispersed repetitive DNA sequences with high similarity. In *D. melanogaster*, ≥2% of meioses yield aberrant chromosomes as a result of ectopic exchange between transposons (Miller *et al.* 2016). The rate of such ectopic exchange undoubtedly fluctuates over time, tracking with the load(s) of transposons of high sequence similarity. The typical evolutionary-demographic history of transposons involves invasion of a new host genome via horizontal transfer (or escape from suppression by the host surveillance system); a burst of proliferation; and eventual silencing upon capture by the host surveillance system (Charlesworth *et al.* 1994; Kidwell and Lisch 2000; Barrón *et al.* 2014). Under this scenario, the risk of ectopic exchange due to any particular transposon will spike with transposon proliferation, as genomes come to harbor a high number of highly similar transposon sequences, and then fade as the sequences of silenced transposons diverge from one another and degenerate. The response to fluctuating selection pressures on crossover rates could be mediated by meiosis genes like *mei-218* and *rec*. Consistent with this hypothesis, in both the *melanogaster* and *obscura* groups, *D. mauritiana* and *D. pseudoobscura* have higher mean rates of crossing over and smaller transposon loads compared to their respective sister species, *D. melanogaster* and *D. persimilis* (Dowsett and Young 1982; True *et al.* 1996; Ortiz-Barrientos *et al.* 2006; Hill and Betancourt 2018). Similarly, within *D. melanogaster*, transposon densities are highest in chromosomal regions that experience little or no crossing over (reviewed in Lee and Langley 2010; Barrón *et al.* 2014). It is important to note, however, that strong alternative models exist in which the presence of transposons favors *increased* rates of crossing over (Charlesworth and Barton 1996). While distinguishing among these hypotheses will be challenging (Charlesworth 2018), it is clear that selfish genetic elements present a ubiquitous, powerful, and perhaps underappreciated source of selection on rates of recombination.
